# Identification of genetic variants of the *IL18R1* gene in association with COPD susceptibility

**DOI:** 10.1080/07853890.2024.2446690

**Published:** 2025-01-23

**Authors:** Jiaoyuan Xu, Meilan Xian, Linhui Huang, Yamei Zheng, Lei Zhang, Jie Zhao, Jie Chen, Siguang Li, Lingsang Lin, Yi Zhong, Zehua Yang, Haihong Wu, Tian Xie, Yipeng Ding

**Affiliations:** a Department of General Practice, Hainan affiliated Hospital of Hainan Medical University, Hainan General Hospital, Haikou, China; b Longbo Health Center of Lingao County, Hainan Province, China; c The 928th Hospital of Joint Logistics Support Force of Chinese People ‘s Liberation Army, Hainan Province, China; d Department of Pulmonary and Critical Care Medicine, Hainan General Hospital, Hainan Affiliated Hospital of Hainan Medical University, Haikou, China

**Keywords:** Chronic obstructive pulmonary disease, case-control study, *IL18R1*, single nucleotide polymorphisms

## Abstract

**Background:**

Although existing studies have identified some genetic loci associated with chronic obstructive pulmonary disease (COPD) susceptibility, many variants remain to be discovered. The aim of this study was to further explore the potential relationship between *IL18R1* single nucleotide polymorphisms (SNPs) and COPD risk.

**Methods:**

Nine hundred and ninety-six subjects were recruited (498 COPD cases and 498 healthy controls). Five candidate SNPs of *IL18R1* were selected and genotyped using MassARRAY iPLEX platform. Logistic regression analysis was performed to assess the association of these SNPs with COPD risk. Multifactor dimensionality reduction (MDR) software was applied to calculate the interaction of SNP-SNP on COPD risk.

**Results:**

*IL18R1* rs9807989 (OR = 0.42, *p* < .001), rs3771166 (OR = 0.40, *p* < .001) and rs6543124 (OR = 0.44, *p* < .001) were associated with the reduced COPD risk, while rs2287037 (OR = 2.71, *p* < .001) and rs2058622 (OR = 2.06, *p* < .001) might be the risk-increasing factor for COPD occurrence in both the overall analysis and subgroup analysis (age, gender, drinking, and smoking). The best multi-locus model was the combination of rs2058622 and rs3771166.

**Conclusion:**

Our study provided a reference and basis for investigating the association of *IL18R1* polymorphisms with COPD risk.

## Introduction

Chronic Obstructive Pulmonary Disease (COPD) is a lung disease characterized by persistent airflow limitation, which is typically caused by the inhalation of harmful particles, particularly tobacco smoke from long-term smoking and air pollution in the environment [1,2]. In 2016, the estimated global prevalence of COPD was 251 million cases, with 113 million of the global cases being in China [3]. A baseline results of an observational study have displayed that the disease burden among Chinese COPD outpatients is high and improved guideline adherence for COPD treatment is needed [4]. At present, risk factors for COPD mainly include active and passive smoking, genetic factors, air pollution, and infection [5,6]. Genome-wide association studies (GWAS) have identified a large number of COPD risk loci [7–10]. By now, more and more genetic loci associated with COPD in the Chinse population have been found, such as *SREK1* rs74794265, *SMAD3* rs36221701, and so on [11,12]. However, these studies are not comprehensive, and there are still a large number of susceptible loci that have not been found. Therefore, it is of great significance to further find genetic markers for COPD and screen susceptible populations.

The interleukin 18 receptor 1 (*IL18R1*) is an interleukin receptor of the immunoglobulin superfamily. The protein encoded by this gene is a cytokine receptor that belongs to the interleukin 1 receptor family. This receptor specifically binds to interleukin 18 (*IL18*) and is essential for *IL18*-mediated signal transduction. *IL18R1* has been reported in some lung diseases. Davinder Kaur et al. [13] have found that *IL18R* may promote airway remodeling in patients with asthma by inducing epithelial cell differentiation, epithelial repair, and changes in the expression of epithelial-mesenchymal transition (EMT) proteins. *IL18R* has been reported to be expressed in alveolar macrophages and bronchoalveolar epithelium of controls, and strongly expressed in interstitial cells of patients with idiopathic pulmonary fibrosis [14]. Fatih Mete et al. have studied the relationship between *IL18R1* gene polymorphisms and childhood asthma in the Turkish population, and discovered that *IL18R1* c. 626-196 G > T (rs3213733) is significantly associated with asthma [15]. Junxian Zhang et al. have shown that the decrease in *IL18R1* mRNA levels caused by rs3755276 may partially mediate the increased susceptibility to tuberculosis [16]. Moreover, Yasuhiko Kitasato et al. have reported IL-18 and IL-18R are involved in the pathogenesis of idiopathic pulmonary fibrosis/usual interstitial pneumonia [14]. A significant association has been found between *IL18R1* gene polymorphisms and asthma [17]. In addition, *IL18R1* SNPs could help identify African-American infants at risk for bronchopulmonary dysplasia [18]. Nevertheless, there are few reports on the association of *IL18R1* single nucleotide polymorphisms (SNPs) with COPD risk so far. The variation in *IL18R1* has been studied in the context of GWAS and no significant association was observed. This may be related to the study population, genetic background and lifestyle habits.

Therefore, we conducted a case-control study including 996 participants in the Chinese Han population. We performed the overall analysis and stratified analysis based on gender, age, smoking, and drinking to analyze the association of *IL18R1* SNPs with COPD risk. This study might provide genetic markers for the prevention, diagnosis and treatment of COPD.

## Materials and methods

### Study participants

In this study, 996 subjects (498 cases and 498 controls) were included in this case-control study to investigate the relationship between *IL18R1* SNPs and COPD risk. The information about subjects including age, gender, smoking, and drinking was obtained from questionnaires. Written informed consent was obtained from all study subjects before a questionnaire interview and biological specimen collection. The experimental protocol was established, according to the ethical guidelines of the Helsinki Declaration and was approved by the Research Ethics Committee of Hainan affiliated Hospital of Hainan Medical University (Med-Eth-Res [2022]312).

Patients with COPD were diagnosed according to the criteria of the Global Initiative for Chronic Obstructive Lung Disease guidelines published in 2017 [19]. COPD patients were included based on the following criteria: patients with COPD should be considered in any patient with dyspnea, sputum production, chronic cough, or exposure to risk factors for the disease. Patients with other major respiratory diseases (tuberculosis, lung cancer, bronchial asthma, and cystic fibrosis) were excluded from this study. Healthy controls were included according to the following criteria: no lung tumor, bronchiectasis, pulmonary fibrosis, tuberculosis and other respiratory diseases.

### DNA extraction and SNP genotyping

Peripheral blood samples (5 mL) from each participant were collected and stored in tubes containing EDTA for subsequent studies. Genomic DNA was extracted from whole blood samples using a GoldMag DNA purification kit (GoldMag Co., Ltd., Xi’an, China), and the concentration and purity of DNA were determined by NanoDrop 2000 (Thermo Scientific, Waltham, MA, USA).

Based on the 1000 Genomes Project (http://www.internationalgenome.org/), we selected candidate *IL18R1* variants with minor allele frequency (MAF) over 5% in the Han Chinese in Beijing, China (CHB). Five SNPs (rs9807989, rs3771166, rs6543124, rs2287037 and rs2058622) of *IL18R1* were selected for this case-control study according to the location, allele frequency and association with the disease. HaploReg v4.1 (https://pubs.broadinstitute.org/mammals/haploreg/haploreg.php) and GTEx Portal database (https://gtexportal.org/home/) with a conservative estimate of α is < 5 × 10^−13^ [20] were applied to predict SNPs potential functions.

All SNPs were genotyped using MassARRAY Nanodispenser (Agena Bioscience, San Diego, CA, USA) and MassARRAY iPLEX platform (Agena Bioscience, San Diego, CA, USA). To control quality, about 5% of the samples were randomly re–genotyped, and the concordance of duplicated genotyping was 100%.

### Statistical analysis

The differences in demographic characteristics (age, gender, smoking and drinking) were analyzed by χ ^2^ test/t-test using SPSS version 25.0 software (SPSS, Chicago, IL, USA). Odds ratios (ORs) and 95% confidence intervals (CIs) were calculated by logistic regression analysis adjusted with age, gender, smoking and drinking using SNPStats (https://snpstats.net/start.htm). The Hardy-Weinberg equilibrium (HWE) test was performed in the control group using the χ^2^ test. Interaction analysis with of *IL18R1* polymorphisms with covariate (age, gender, smoking and drinking) was performed using SNPStats. Multifactor dimensionality reduction (MDR) software was then applied to calculate the interaction of SNP-SNP on COPD risk using MDR 3.0.2 (https://sourceforge.net/projects/mdr/). Using this method, multilocus genotypes are classified into high-risk and low-risk groups, effectively reducing the genotype predictors from n dimensions to one dimension. The new, one-dimensional multilocus genotype variable is evaluated for its ability to classify and predict disease status through cross-validation (CV). The MDR method is model-free, in that it does not assume any particular genetic model. Its method of determining high-risk or low-risk groups is ad hoc—in the sense that it classifies cells, defined by a combination of multilocus genotypes, into high-risk or low-risk groups based on a simple comparison of the ratios of the number of cases and controls [21–23]. D’ and R^2^ values for pairwise linkage disequilibrium (LD) plots were generated by Haploview software (version 4.2), and the association of *IL18R1* haplotypes with COPD risk was evaluated by logistic regression model. Finally, the STRING database and online mapping platform (https://cloud.oebiotech.cn/task/) were utilized to carry out protein-protein interaction (PPI) network and Kyoto Encyclopedia of Genes and Genomes (KEGG) pathway enrichment analysis. A *p* value less than 0.05 for logistic regression models was considered statistically significant, and a *p* < 0.05/5 for logistic regression models was deemed significant after Bonferroni correction.

## Results

### Study population

In this study, the average age of COPD patients (including 328 males (65.9%) and 170 females (34.1%)) was 70.9 ± 10.2 years. The average age of controls (including 322 males (64.7%) and 176 females (35.3%)) was 65.9 ± 5.6 years. The basic statistical information was shown in Table 1. A statistic difference in age (*p* < 0.001) between the two groups were observed. There was no apparent discrepancy in smoking (*p* = 0.848), gender (*p* = 0.690) and drinking (*p* = 0.800) between cases and controls.

**Table 1.** Characteristics of patients with chronic obstructive pulmonary disease and healthy.

Characteristics	Cases (*n* = 498)	Controls (*n* = 498)	*p* value
Age (years, Mean ± SD)	70.9 ± 10.2	65.9 ± 5.6	< .001 ^a^
> 60	424 (85.1%)	441 (88.6%)	
≤ 60	74 (14.9%)	57 (11.4%)	
Gender			.690^b^
Male	328 (65.9%)	322 (64.7%)	
Female	170 (34.1%)	176 (35.3%)	
Smoking status			.848^b^
Smoking	218 (43.8%)	221 (44.4%)	
Non – smoking	280 (56.2%)	277 (55.6%)	
Drinking status			.800^b^
Drinking	251 (50.4%)	255 (51.2%)	
Non – drinking	247 (49.6%)	243 (48.8%)	
BMI			<.001 ^a^
<24 kg/m^2^	435 (87.3%)	152 (30.5%)	
≥ 24 kg/m^2^	50 (10.0%)	152 (30.5%)	
Missing	13 (2.6%)	194 (39.0%)	
FVC, L	1.64 ± 0.65		
FEV1, L	1.25 ± 0.56		
FEV1/FVC, %	60.49 ± 33.95		
GLOD stage			
I	98 (19.7%)		
II	84 (16.9%)		
III	19 (3.8%)		
IV	55 (11.0%)		
Missing	242 (48.6%)		
Disease staging			
Acute exacerbation	244 (49.0%)		
Remission period	229 (46.0%)		
Missing	55 (5.0%)		
Smoking history for smokers (*N* = 218)			
≤ 30 years	77 (35.3%)		
31–50 years	108 (49.5%)		
> 50 years	33 (15.1%)		
Passive smoking history for non-smokers (*N* = 280)			
No	189 (67.5%)		
Yes	61 (21.8%)		
Missing	30 (10.7%)		
Comorbidities			
No	298 (59.8%)		
Yes	177 (35.5%)		
Missing	23 (4.6%)		
Cough			
No	12 (2.4%)		
Yes	474 (95.2%)		
Missing	12 (2.4%)		
Expectoration			
No	49 (9.8%)		
Yes	437 (87.8%)		
Missing	12 (2.4%)		
Gasp			
No	334 (67.1%)		
Yes	152 (30.5%)		
Missing	12 (2.4%)		
Dyspnea			
No	421 (84.5%)		
Yes	65 (13.1%)		
Missing	12 (2.4%)		
Inhalation dust			
No	440 (88.4%)		
Yes	46 (9.2%)		
Missing	12 (2.4%)		

*P*^a^ and *P*^b^ values were calculated by independent sample t-test and Pearson’s χ^2^ test, respectively.

*p* < .05 indicates statistical significance.

### Information about candidate SNPs

The details of SNPs in *IL18R1* were summarized in Table  2. Five candidate genetic loci (rs9807989 C/T, rs2287037 T/C, rs2058622 G/A, rs3771166 A/G and rs6543124 A/T) were genotyped and all of them were located on chromosome 2. The call rate of these SNPs was > 99.0%. In addition, they were consistent with HWE (*p* > .05). Based on the NCBI database (https://www.ncbi.nlm.nih.gov/variation/view/), these SNPs were located in the upstream or intron 1 region of the *IL18R1* gene. Based on Haploreg v4.1, these SNPs might be related to the regulation of NHGRI/EBI GWAS hits, motif changes, selected eQTL hits and GRASP QTL hits. Based on the GTEx Portal database, the genotypes of rs9807989 (*p* = 9.4e-20), rs3771166 (*p* = 9.69e-21), and rs6543124 (*p* = 2.5e-20) were related to the mRNA expression in the lung tissue (Supplementary Figure 1).

**Table 2.** The basic information about the selected SNPs of *IL18R1.*

Gene	SNP ID	Chr: position	Alleles (A/B)	MAF		Call rate	HWE (*P* value)	dbSNP database	Haploreg v4.1
				Cases	Controls				
IL18R1	rs9807989	2:102354740	C/T	0.056	0.125	99.4%	0.095	Intron 1	Motifs changed; NHGRI/EBI GWAS hits; Selected eQTL hits
IL18R1	rs2287037	2:102362568	T/C	0.564	0.323	99.4%	0.607	Intron 1	GRASP QTL hits; Selected eQTL hits
IL18R1	rs2058622	2:102368964	G/A	0.622	0.444	99.4%	0.274	Intron 1	Motifs changed; GRASP QTL hits; Selected eQTL hits
IL18R1	rs3771166	2:102369762	A/G	0.058	0.133	99.5%	0.240	Intron 1	Motifs changed; NHGRI/EBI GWAS hits; Selected eQTL hits
IL18R1	rs6543124	2:102370999	A/T	0.051	0.109	100.0%	0.820	Intron 1	Motifs changed; Selected eQTL hits

SNP: single nucleotide polymorphism; MAF: minor allele frequency; HWE: Hardy-Weinberg equilibrium.

### Association of IL18R1 polymorphisms with COPD risk

**Figure 1.**
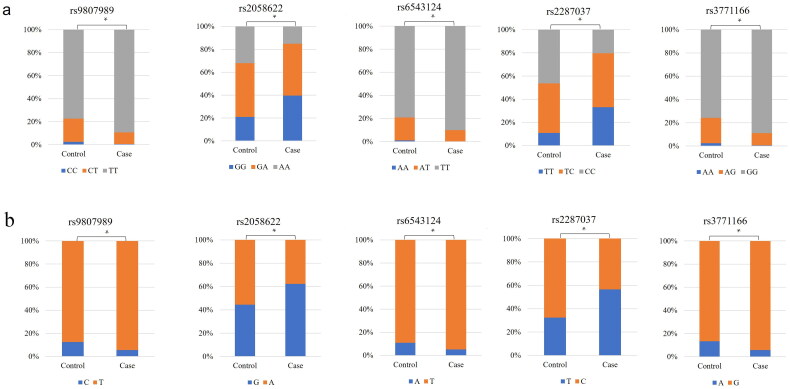
The histogram plot for the genotype (a) and allele (b) frequency distribution of *IL18R1* polymorphisms between healthy individuals (the control group) and COPD patients (the case group). *p < .05 represent statistical significance of the genotype or allele frequencies distribution between two groups. Different colors represent different genotypes or alleles.

There were significant differences (*p* < .01) in the genotype and allele frequency distribution of *IL18R1* polymorphisms between healthy individuals and COPD patients (Figure 1 and Table 3).

**Table 3.** Analysis of the association between susceptibility of chronic obstructive pulmonary disease and single nucleotide polymorphism of *IL18R1.*

SNP	Model	Genotype	Case	Control	Without adjusted		With adjusted	
					OR (95% CI)	*P* ^1^	OR (95% CI)	*P* ^2^
rs9807989	Allele	T	940 (94.4%)	861 (87.5%)			1.00	
		C	56 (5.6%)	123 (12.5%)			0.42 (0.30–0.58)	<.001*
	Genotype	TT	445 (89.4%)	381 (77.4%)	1.00		1.00	
		CT	50 (10%)	99 (20.1%)	0.43 (0.30–0.62)	<0.001*	0.42 (0.29–0.62)	<.001*
		CC	3 (0.6%)	12 (2.4%)	0.21 (0.06–0.76)	0.018*	0.17 (0.05–0.64)	.008*
	Dominant	TT	445 (89.4%)	381 (77.4%)	1.00		1.00	
		CC-CT	53 (10.6%)	111 (22.6%)	0.41 (0.29–0.58)	<0.001*	0.39 (0.27–0.57)	<.001*
	Recessive	CT-TT	495 (99.4%)	480 (97.6%)	1.00		1.00	
		CC	3 (0.6%)	12 (2.4%)	0.24 (0.07–0.86)	0.029*	0.20 (0.05–0.72)	.014*
	Log-additive	/			0.44 (032–0.61)	<0.001*	0.42 (0.30–0.59)	<.001*
rs2287037	Allele	C	434 (43.6%)	666 (67.7%)			1.00	
		T	562 (56.4%)	318 (32.3%)			2.71 (2.26–3.26)	<.001*
	Genotype	CC	101 (20.3%)	228 (46.3%)	1.00		1.00	
		TC	232 (46.6%)	210 (42.7%)	2.49 (1.85–3.37)	<0.001*	2.31 (1.70–3.16)	<.001*
		TT	165 (33.1%)	54 (11%)	6.90 (4.69–10.15)	<0.001*	7.39 (4.91–11.13)	<.001*
	Dominant	CC	101 (20.3%)	228 (46.3%)	1.00		1.00	
		TT-TC	397 (79.7%)	264 (53.7%)	3.40 (2.56–4.50)	<0.001*	3.26 (2.44–4.36)	<.001*
	Recessive	TC-CC	333 (66.9%)	438 (89%)	1.00		1.00	
		TT	165 (33.1%)	54 (11%)	4.02 (2.86–5.64)	<0.001*	4.56 (3.17–6.556)	<.001*
	Log-additive	/			2.61 (2.16–3.15)	<0.001*	2.66 (2.18–3.24)	<.001*
rs2058622	Allele	A	376 (37.8%)	547 (55.6%)			1.00	
		G	620 (62.2%)	437 (44.4%)			2.06 (1.73–2.47)	<.001*
	Genotype	AA	75 (15.1%)	158 (32.1%)	1.00		1.00	
		GA	226 (45.4%)	231 (47%)	2.06 (1.48–2.89)	<0.001*	1.89 (1.35–2.67)	<.001*
		GG	197 (39.6%)	103 (20.9%)	4.03 (2.80–5.80)	<0.001*	3.94 (2.70–5.77)	<.001*
	Dominant	AA	75 (15.1%)	158 (32.1%)	1.00		1.00	
		GG-GA	423 (84.9%)	334 (67.9%)	2.67 (1.96–3.64)	<0.001*	2.50 (1.81–3.44)	<.001*
	Recessive	GA-AA	301 (60.4%)	389 (79.1%)	1.00		1.00	
		GG	197 (39.6%)	103 (20.9%)	2.47 (1.87–3.28)	<0.001*	2.57 (1.91–3.46)	<.001*
	Log-additive	/			2.00 (1.67–2.40)	<0.001*	1.99 (1.65–2.41)	<.001*
rs3771166	Allele	G	938 (94.2%)	858 (86.7%)			1.00	
		A	58 (5.8%)	132 (13.3%)			0.40 (0.29–0.56)	<.001**
	Genotype	GG	443 (89%)	375 (75.8%)	1.00		1.00	
		AG	52 (10.4%)	108 (21.8%)	0.41 (0.29–0.58)	<0.001*	0.40 (0.27–0.57)	<.001*
		AA	3 (0.6%)	12 (2.4%)	0.21 (0.06–0.76)	0.017*	0.17 (0.05–0.63)	<.001*
	Dominant	GG	443 (89%)	375 (75.8%)	1.00		1.00	
		AA-AG	55 (11%)	120 (24.2%)	0.39 (0.27–0.549)	<0.001*	0.37 (0.26–0.53)	<.001*
	Recessive	AG-GG	495 (99.4%)	483 (97.6%)	1.00		1.00	
		AA	3 (0.6%)	12 (2.4%)	0.24 (0.07–0.87)	0.030**	0.20 (0.05–0.72)	.014*
	Log-additive	/			0.42 (0.30–0.58)	<0.001	0.40 (0.29–0.56)	<.001*
rs6543124	Allele	T	945 (94.9%)	887 (89.1%)			1.00	
		A	51 (5.1%)	109 (10.9%)			0.44 (0.31–0.62)	<.001*
	Genotype	TT	448 (90%)	394 (79.1%)	1.00		1.00	
		AT	49 (9.8%)	99 (19.9%)	0.44 (0.30–0.63)	<0.001*	0.41 (0.28–0.60)	<.001*
		AA	1 (0.2%)	5 (1%)	0.18 (0.02–1.51)	0.113	0.13 (0.01–1.16)	.067
	Dominant	TT	448 (90%)	394 (79.1%)	1.00		1.00	
		AA-AT	50 (10%)	104 (20.9%)	0.42 (0.29–0.61)	<0.001*	0.40 (0.27–0.58)	<.001*
	Recessive	AT-TT	497 (99.8%)	493 (99%)	1.00		1.00	
		AA	1 (0.2%)	5 (1%)	0.20 (0.02–1.70)	0.141	0.15 (0.02–1.32)	.087
	Log-additive	/			0.43 (0.31–0.61)	<0.001*	0.41 (0.28–0.58)	<.001*

SNP: single nucleotide polymorphism; OR: odds ratio; CI: confidence interval.

*P*^1^ values were calculated by logistic regression analysis without adjustment.

*P*^2^ values were calculated by logistic regression analysis with adjustment by age, gender, smoking and drinking.

* *p* < .05 represent statistical significance.

As shown in Table 3, rs9807989, rs3771166 and rs6543124 might be protective factors against COPD. Specifically, rs9807989 was associated with the reduced COPD risk in the allele (OR = 0.42, *p* < .001), genotype (OR = 0.42, *p* < .001, and OR = 0.17, *p* = .008), dominant (OR = 0.39, *p* < .001), recessive (OR = 0.20, *p* = .014) and additive (OR = 0.42, *p* < .001) models. Rs3771166 was related to the lower COPD susceptibility under the allele (OR = 0.40, *p* < .001), genotype (OR = 0.40, *p* < .001, and OR = 0.17, *p* < .001), dominant (OR = 0.37, *p* < .001), recessive (OR = 0.20, *p* = .014) and additive (OR = 0.40, *p* < .001) models. Rs6543124 was associated with the lower COPD risk under the allele (OR = 0.44, *p* < .001), genotype (OR = 0.41, *p* < .001), dominant (OR = 0.40, *p* < .001), and additive (OR = 0.41, *p* < .001) models. However, rs2287037 (allele: OR = 2.71, *p* < .001; genotype: OR = 2.31, *p* < .001, and OR = 7.39, *p* < .001; dominant: OR = 3.26, *p* < .001; recessive: OR = 4.56, *p* < .001; additive: OR = 2.66, *p* < .001) and rs2058622 (allele: OR = 2.06, *p* < .001; genotype: OR = 1.89, *p* < .001, and OR = 3.94, *p* < .001; dominant: OR = 2.50, *p* < .001; recessive: OR = 2.57, *p* < .001; additive: OR = 1.99, *p* < .001) might be risk-increasing factors for COPD in multiple models. The significance of the risk-reducing association of rs9807989, rs3771166 and rs6543124 with COPD and the risk-increasing relationship of rs2287037 and rs2058622 to COPD susceptibility still existed after Bonferroni correction (*p* < 0.05/5). Supplementary Table 1 diaplayed the interaction of *IL18R1* polymorphisms with covariate (age, gender, smoking and drinking). The results displayed that the interaction of rs13015714, rs2287037 and rs2058622 with covariate age was found. Moreover, the interactions of rs9807989 and rs3771166 with gender was observed.

### Stratification analysis

**Figure 2.**
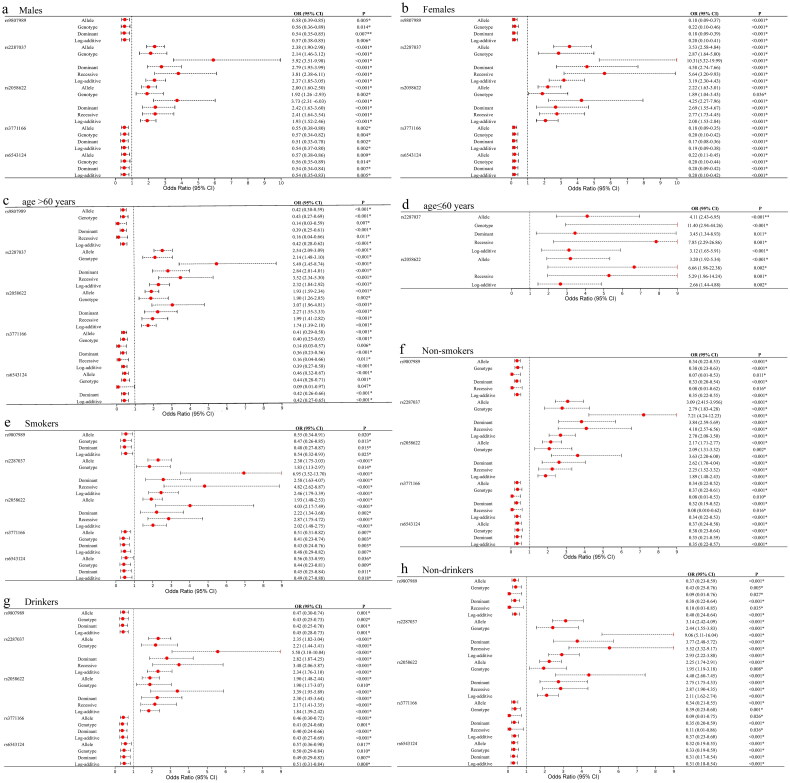
Forest Map for the stratification analysis of various confounding factors such as gender (a,b), age (c,d), smoking (e,f), and alcohol consumption (g,h). The red dots denote the SNP/model associated with an increased risk of COPD, green dots represent the SNP/model related to a decreased risk, and black dots indicate the SNP/model with no significant association to COPD risk.

This study conducted the stratification analysis of various confounding factors such as gender, age, smoking, and alcohol consumption to explore the relationship between *IL18R1* polymorphisms and COPD susceptibility (Figure 2).

#### Gender

*IL18R1* rs9807989, rs3771166 and rs6543124 were associated with decreased COPD risk, while rs2287037 and rs2058622 were related to the increased COPD risk in males and females under multiple genetic models (Supplementary Table 1).

#### Age

Rs9807989, rs3771166 and rs6543124 were related to the reduced susceptibility to COPD in subjects aged > 60 years under genetic models (Supplementary Table 2). However, rs2287037 and rs2058622 were related to the increased risk of COPD in participants aged > 60 years and subjects aged ≤ 60 years.

#### Drinking and smoking

For smoking, participants were classified as non-smokers (never) and smokers (including former or current smokers). Rs9807989, rs3771166 and rs6543124 might be associated with the lower COPD risk, whereas rs2287037 and rs2058622 could contribute to the higher COPD risk in smokers, non-smokers, drinkers and non-drinkers (Supplementary Tables 3 and 4).

### MDR analysis

**Figure 3.**
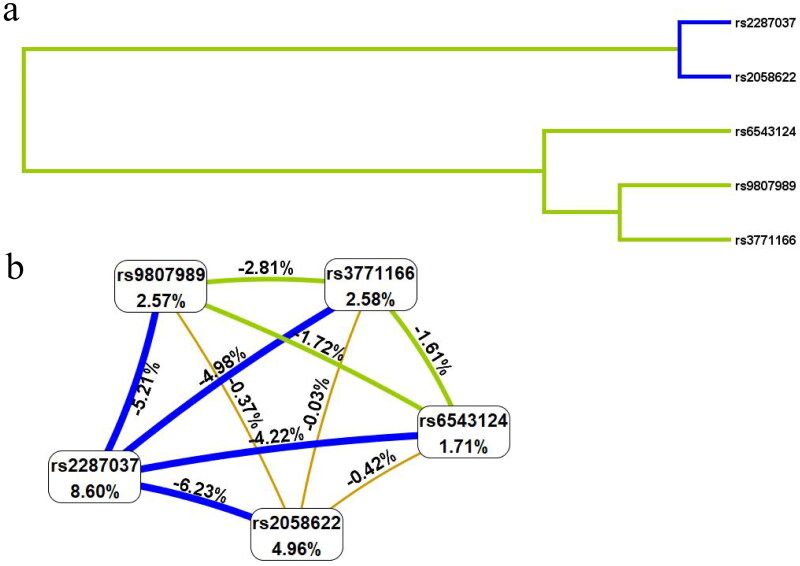
SNP-SNP Interaction dendrogram (a) and Fruchterman-Reingold (b). Green and blue represent redundancy or association. The longer the branches or the greater the distance between nodes, the less similar the groups. The shorter the branches or the smaller the distance between nodes, the more similar the groups (a). Values in nodes represent the information gains of individual attribute (main effects). Values between nodes are information gains of each pair of attributes (interaction effects, b).

MDR was used to predict and assess SNP-SNP interactions. The dendrogram (Figure 3a) showed that the loci with strong interactions were close together on the branches, while the loci with weak interactions were far apart from each other. As shown in Table 4 and Figure 3b, the most single-locus influential attributor for COPD risk was rs2287037 (testing balanced accuracy (TBA): 0.634 and good cross-validation consistency (CVC): 10/10) with the information gain of 8.60%, and the best multi-locus model was the combination of rs2058622 and rs3771166 (TBA: 0.657 and a good CVC: 10/10) with the information gain of −0.03%.

**Table 4.** *IL18R1* SNP–SNP Interaction models analyzed by the MDR method.

Model	Training Bal. Acc.	Testing Bal. Acc.	CVC	*p*
rs2287037	0.634	0.634	10/10	<.001*
rs2058622, rs3771166	0.658	0.657	10/10	<.001*
rs2287037, rs2058622, rs6543124	0.665	0.656	10/10	<.001*
rs9807989, rs2287037, rs2058622, rs6543124	0.667	0.652	7/10	<.001*
rs9807989, rs2287037, rs2058622, rs3771166, rs6543124	0.668	0.648	10/10	<.001*

MDR: multifactor dimensionality reduction; Bal. Acc.: balanced accuracy; CVC: cross-validation consistency; OR: odds ratio; 95% CI: 95% confidence interval.

*p* values were calculated by logistic regression analysis.

* *p* < .01 represent statistical significance.

### Association between IL18R1 haplotypes and the risk of COPD

**Figure 4.**
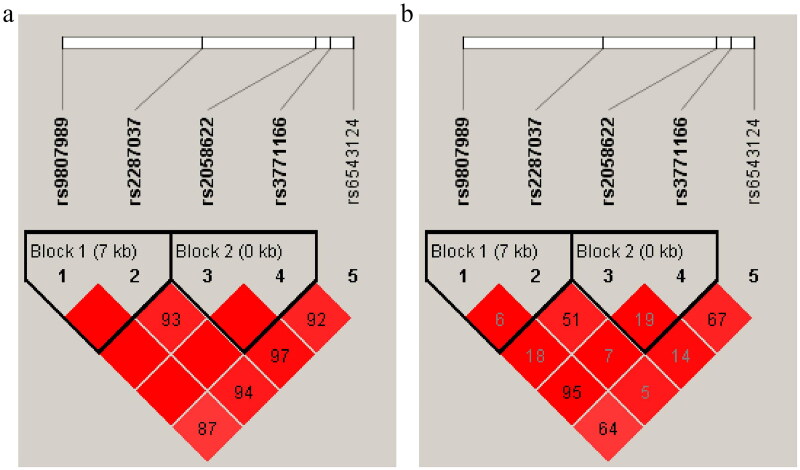
Haplotype block map for eight SNPs in the *IL18R1* gene. (a) The numbers inside the diamonds indicate the D′ for pairwise analyses. (b) The numbers inside the diamonds indicate the R^2^ for pairwise analyses.

Moreover, LD and haplotype analysis was performed to estimate the association between *IL18R1* haplotypes and the risk of COPD. As shown in Figure 4, the high LD blocks were composed of rs9807989 and rs2287037 for Block 1 including TC, TT and CC haplotypes, and rs2058622 and rs3771166 for Block 2 including AG, GG and GA haplotypes. Furthermore, the haplotype frequency distribution was shown in Table 5. We noted that T_rs9807989_ T_rs2287037_ (OR = 2.32, *p* < .001) and G_rs2058622_ G_rs3771166_ (OR = 2.33, *p* < .001) haplotypes were associated with the increased COPD risk.

**Table 5.** Haplotype analysis for the effect of *IL18R1* haplotypes on the risk of COPD.

Haplotype	Frequency		Crude analysis		Adjusted analysis	
	Control	Case	OR (95% CI)	*p*	OR (95% CI)	*p*
Block 1 (rs9807989 and rs2287037)						
TC	0.380	0.552	1		1	
TT	0.564	0.323	2.45 (2.02–2.99)	<.0001*	2.32 (1.83–2.94)	<.0001*
CC	0.056	0.125	0.70 (0.50–0.99)	.042*	0.79 (0.52–1.20)	.270
Block 2 (rs2058622 and rs3771166)						
AG	0.378	0.557	1		1	
GG	0.564	0.309	2.50 (2.06–3.04)	<.0001*	2.33 (1.84–2.95)	<.0001*
GA	0.058	0.134	0.68 (0.48–0.95)	.025*	0.74 (0.49–1.12)	.150

SNP: Single nucleotide polymorphism; OR: Odds ratio; 95% CI: 95% confidence interval.

*p*-values were calculated by logistic regression analysis with adjustments for age, gender, smoking, and drinking.

* *p* < .05 represent statistical significance.

### PPI network and KEGG pathway analysis

**Figure 5.**
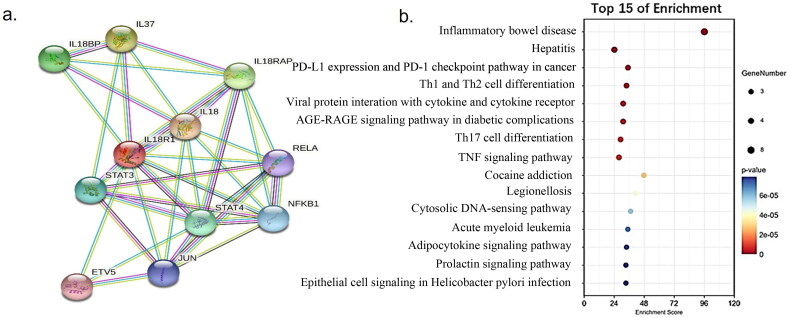
PPI (a) And KEGG (b) analysis for *IL18R1* related genes. Nodes in the network represent proteins, and lines between nodes represent interactions between proteins (a). The depth of the color may represent significance, the size of the bubble, and the number of genes involved in that pathway (b).

The STRING database and online mapping platform (Oebiological Cloud Platform) (https://cloud.oebiotech.cn/task/) were respectively used to construct a PPI network and KEGG pathway analysis of *IL18R1. IL18R1* might interact with *IL18BP* (interleukin 18 binding protein), *STAT4* (signal transducer and activator of transcription 4), *STAT3* (signal transducer and activator of transcription 3), *IL18*, *Jun* and so on (Figure 5a). In addition, *IL18R1* might be mainly involved in inflammatory bowel disease, PD-L1 (programmed death-ligand 1) expression, and hepatitis B, PD-1 (programmed cell death protein-1) checkpoint pathway in cancer and Th1/Th2 cell differentiation (Figure 5b).

## Discussion

Here, our study analyzed the genotypes of five SNPs (rs9807989, rs3771166, rs6543124, rs2287037 and rs2058622) of *IL18R1* and their association with COPD susceptibility. Our finding displayed that *IL18R1* rs9807989, rs3771166, and rs6543124 were associated with reduced COPD risk, while rs2287037 and rs2058622 might be the risk-increasing factor for COPD occurrence.

*IL18R1* is located on chromosome 2 and is an interleukin receptor of the immunoglobulin superfamily. Previous studies displayed that *IL18R1* plays an important role in lung disease [17,18]. However, relatively few studies have explored the relationship between *IL18R1* polymorphisms and COPD risk. Five SNPs (rs9807989, rs3771166, rs6543124, rs2287037 and rs2058622) are located in the upstream and intron of the *IL-18R1* gene on chromosome 2, separately. Previous studies have shown that rs9807989 is related to the susceptibility to severe asthma [24]. Wan et al. [25] have pointed out that rs3771166 has a protective effect in a cohort of patients with severe asthma. Currently, there are no reports about the rs6543124 locus. Haralambieva Iana et al. [26] have found that rs2287037 is related to antibody levels in African Americans (*p* ≤ .025) and European (*p* ≤ .016). Some researchers has found that rs2287037 is associated with lumbar disc degeneration and allergic rhinitis [27,28]. Soderquest Katrina et al. [29] have reported that rs2058622 contributes to mucosal autoimmune disease by altering the binding of lineage specific transcription factors and gene expression. These studies have shown that *IL18R1* gene polymorphisms are associated with a variety of inflammatory diseases, but there are few reports on its association with the occurrence of COPD. Our finding displayed that *IL18R1* rs9807989, rs3771166 and rs6543124 might have the lower COPD risk, while rs2287037 and rs2058622 might have the higher COPD risk. HaploReg v4.1 [30] displayed that these five SNPs of *IL18R1* might participate in the regulation of NHGRI/EBI GWAS hits, changed motifs, and selected eQTL hits. Moreover, the GTEx Portal database displayed the genotypes of these five SNPs were related to the mRNA expression of IL18R1 in the lung tissue. Therefore, we speculated that *IL18R1* polymorphisms might influence the occurrence of COPD by affecting *IL18R1* gene expression. However, this hypothesis requires experimental verification.

Previous studies have shown that age, gender, smoking, and alcohol consumption are risk factors for COPD. A systematic analysis for the Global Burden of Disease Study 2015 reported a global age-standardized prevalence of 3.2% [male]/2.0% [female] for COPD [31]. Moreover, the prevalence of age-related chronic diseases like COPD is also increasing along with morbidity, mortality, and a greater degree of disability [32]. Exposure to inhaled noxious particles, notably tobacco smoke and pollutants was a risk factor for COPD [33]. Alcohol consumption is also a risk factor for COPD [34]. Alcohol ingestion disrupts alveolar epithelial barrier function by activation of macrophage-derived transforming growth factor beta1 [35]. A heavy consumption of alcohol was associated with an increased risk of exacerbation of COPD but was confounded by tobacco use, which is strongly associated with alcohol consumption [36]. Therefore, the stratification analysis was preformed to explore the relationship between the *IL18R1* polymorphisms and COPD susceptibility. Our study revealed that these five SNPs were significantly associated with COPD risk in participants in males, females, subjects with age > 60 years, smokers, non-smokers, drinkers and non-drinkers under the allele, genotype, dominant, recessive and additive models.

The potential mechanisms of *IL18R1* SNPs in COPD development have not been studied. On this basis, we preliminarily explored the protein targets and related pathways that interacted with *IL18R1*, with a view to discovering the possible pathological mechanisms by which five-candidate SNPs affect COPD risk. One research has found that *Stat4* is over-expressed in Th1 cells [33], which is an important part of the *IL18R1* locus [37]. *IL-18* is a cytokine of the *IL-1* family, and elevated *IL-18* levels have been found in many chronic inflammatory diseases [38,39]. Therefore, we speculated that these five candidate polymorphisms might regulate related pathways such as Th1 and Th2 cell differentiation and other pathways, and thus played a role in the progression of COPD.

It was worth emphasizing that there are still limitations in this study. Firstly, the sample size should be further expanded and the training samples should be collected to validate our results in the future. Secondly, we will collect Bronchoalveolar Lavage (BAL) samples to confirm our finding in subsequent researches. Thirdly, it was still necessary to further study how *IL18R1* polymorphisms affect the potential pathogenesis of COPD. Fourth, the information about the clinical index about pulmonary function tests (spirometry and DLCO) for both groups and environmental exposure including air pollution, occupational exposure (such as long-term exposure to dust or harmful chemicals), and biomass-burning smoke ‘BBS’ exposure of COPD patients was incomplete, it was not explored in the article. In the follow-up study, we will expand the sample size and complete the data to confirm our findings. Despite the above limitations, our current research provided new insights into the relationship between the *IL18R1* gene and COPD. In subsequent studies, we will focus on the effect of exposure to harmful substances on the occurrence of COPD, and explore this effect by collecting larger samples and rigorously measuring exposure to harmful substances in cases and controls.

## Conclusion

Taken together, our results suggested that candidate *IL18R1* SNPs were strongly related to COPD risk. *IL18R1* rs9807989, rs3771166 and rs6543124 were associated with reduced COPD risk, while rs2287037 and rs2058622 might be the risk-increasing factor for COPD occurrence. Our study provided a reference and basis for investigating the association of *IL18R1* polymorphisms with COPD risk.

## Supplementary Material

Supplemental Material

## Data Availability

The data that support the findings of this study are available from the corresponding author, YP Ding, upon reasonable request.
