# Supplementary material for: Identification of genetic variants of the IL18R1 gene in association with COPD susceptibility
Source: Ann Med. 2025 Jan 23;57(1):2446690. doi: 10.1080/07853890.2024.2446690 (PMC11758794; doi:10.1080/07853890.2024.2446690)
Supplement: Supplemental Material [file IANN_A_2446690_SM0540.zip › Suppl/Suppl_Tables clean.docx]

**Suppl_Table 1 Interaction analysis with of *IL18R1* polymorphisms with covariate (age, gender, smoking and drinking)**

| SNP ID | Interaction analysis with covariate age | | | Interaction analysis with covariate gender | | |
| --- | --- | --- | --- | --- | --- | --- |
|  | Codominant | Dominant | Recessive | Codominant | Dominant | Recessive |
| rs9807989 | 0.770 | 0.800 | NA | 0.052 | **0.035** | 0.100 |
| rs13015714 | **0.003** | **0.001** | 0.840 | 0.790 | 0.970 | 0.370 |
| rs2287037 | **0.026** | 0.380 | **0.007** | 0.350 | 0.100 | 0.770 |
| rs2058622 | **0.016** | **0.005** | 0.730 | 0.940 | 0.870 | 0.620 |
| rs3771166 | 0.350 | 0.370 | NA | **0.049** | **0.032** | 0.100 |
| rs6543124 | 0.260 | 0.260 | NA | 0.270 | 0.130 | 0.480 |
| SNP ID | Interaction analysis with covariate smoking | | | Interaction analysis with covariate drinking | | |
|  | Codominant | Dominant | Recessive | Codominant | Dominant | Recessive |
| rs9807989 | 0.420 | 0.200 | 0.620 | 0.380 | 0.600 | 0.160 |
| rs13015714 | 1.000 | 0.970 | 0.860 | 0.600 | 0.360 | 0.550 |
| rs2287037 | 0.430 | 0.130 | 0.650 | 0.760 | 0.370 | 0.950 |
| rs2058622 | 0.940 | 0.840 | 0.620 | 0.800 | 0.570 | 0.810 |
| rs3771166 | 0.490 | 0.240 | 0.620 | 0.330 | 0.380 | 0.160 |
| rs6543124 | 0.095 | 0.190 | 0.057 | 0.140 | 0.110 | 0.170 |

SNP, Single nucleotide polymorphism.

Bold *p* < 0.05 respects the data is statistically significant.

**Suppl_Table 2 The SNPs** **of *IL18R1* associated with susceptibility of chronic obstructive pulmonary disease in the subgroup tests (Gender)**

| SNP | Model | Genotype | Male | | | | Female | | | |
| --- | --- | --- | --- | --- | --- | --- | --- | --- | --- | --- |
|  |  |  | Case | Control | OR (95% CI) | *P* | Case | Control | OR (95% CI) | *P* |
| rs9807989 | Allele | T | 610 | 559 | 1.00 |  | 330 | 302 | 1.00 |  |
|  |  | C | 46 | 73 | 0.58 (0.39-0.85) | 0.005^*^ | 10 | 50 | 0.18 (0.09-0.37) | <0.001^*^ |
|  | Genotype | TT | 285 | 248 | 1.00 |  | 160 | 133 | 1.00 |  |
|  |  | CT | 40 | 63 | 0.56 (0.36-0.89) | 0.014^*^ | 10 | 36 | 0.22 (0.10-0.46) | <0.001^*^ |
|  |  | CC | 3 | 5 | 0.34 (0.08-1.54) | 0.163 | 0 | 7 | / | / |
|  | Dominant | TT | 285 | 248 | 1.00 |  | 160 | 133 | 1.00 |  |
|  |  | CC-CT | 43 | 68 | 0.54 (0.35-0.85) | 0.007^**^ | 10 | 43 | 0.18 (0.09-0.39) | <0.001^*^ |
|  | Recessive | CT-TT | 325 | 311 | 1.00 |  | 170 | 169 | 1.000 |  |
|  |  | CC | 3 | 5 | 0.38 (0.08-1.68) | 0.201 | 0 | 7 | / | / |
|  | Log-additive | / | / | / | 0.57 (0.38-0.85) | 0.006^*^ | / | / | 0.20 (0.10-0.41) | <0.001^*^ |
| rs2287037 | Allele | C | 308 | 431 | 1.00 |  | 126 | 235 | 1.00 |  |
|  |  | T | 348 | 205 | 2.38 (1.90-2.98) | <0.001^*^ | 214 | 113 | 3.53 (2.58-4.84) | <0.001^*^ |
|  | Genotype | CC | 74 | 145 | 1.00 |  | 27 | 83 | 1.00 |  |
|  |  | TC | 160 | 141 | 2.14 (1.46-3.12) | <0.001^*^ | 72 | 69 | 2.87 (1.64-5.00) | <0.001^*^ |
|  |  | TT | 94 | 32 | 5.92 (3.51-9.98) | <0.001^*^ | 71 | 22 | 10.31(5.32-19.99) | <0.001^*^ |
|  | Dominant | CC | 74 | 145 | 1.00 |  | 27 | 83 | 1.00 |  |
|  |  | TT-TC | 254 | 173 | 2.79 (1.95-3.99) | <0.001^*^ | 143 | 91 | 4.58 (2.74-7.66) | <0.001^*^ |
|  | Recessive | TC-CC | 234 | 286 | 1.00 |  | 99 | 152 | 1.00 |  |
|  |  | TT | 94 | 32 | 3.81 (2.38-6.11) | <0.001^*^ | 71 | 22 | 5.64 (3.20-9.93) | <0.001^*^ |
|  | Log-additive | / | / | / | 2.37 (1.85-3.05) | <0.001^*^ | / | / | 3.19 (2.30-4.43) | <0.001^*^ |
| rs2058622 | Allele | A | 260 | 360 | 1.00 |  | 116 | 187 | 1.00 |  |
|  |  | G | 396 | 274 | 2.00 (1.60-2.50) | <0.001^*^ | 224 | 163 | 2.22 (1.63-3.01) | <0.001^*^ |
|  | Genotype | AA | 52 | 104 | 1.00 |  | 23 | 54 | 1.00 |  |
|  |  | GA | 156 | 152 | 1.92 (1.26 -2.93) | 0.002^*^ | 70 | 79 | 1.89 (1.04-3.43) | 0.036^*^ |
|  |  | GG | 120 | 61 | 3.73 (2.31 -6.03) | <0.001^*^ | 77 | 42 | 4.25 (2.27-7.96) | <0.001^*^ |
|  | Dominant | AA | 52 | 104 | 1.00 |  | 23 | 54 | 1.00 |  |
|  |  | GG-GA | 276 | 213 | 2.42 (1.63-3.60) | <0.001^*^ | 147 | 121 | 2.69 (1.55-4.67) | <0.001^*^ |
|  | Recessive | GA-AA | 208 | 256 | 1.00 |  | 93 | 133 | 1.00 |  |
|  |  | GG | 120 | 61 | 2.41 (1.64-3.54) | <0.001^*^ | 77 | 42 | 2.77 (1.73-4.45) | <0.001^*^ |
|  | Log-additive | / | / | / | 1.93 (1.52-2.46) | <0.001^*^ | / | / | 2.08 (1.53-2.84) | <0.001^*^ |
| rs3771166 | Allele | G | 260 | 360 | 1.00 |  | 330 | 300 | 1.00 |  |
|  |  | A | 396 | 274 | 0.55 (0.38-0.80) | 0.002^*^ | 10 | 52 | 0.18 (0.09-0.35) | <0.001^*^ |
|  | Genotype | GG | 283 | 244 | 1.00 |  | 160 | 131 | 1.00 |  |
|  |  | AG | 42 | 70 | 0.57 (0.34-0.82) | 0.004^*^ | 10 | 38 | 0.20 (0.10-0.42) | <0.001^*^ |
|  |  | AA | 3 | 5 | 0.34 (0.08-1.52) | 0.158 | 0 | 7 | / | / |
|  | Dominant | GG | 283 | 244 | 1.00 |  | 160 | 131 | 1.00 |  |
|  |  | AA-AG | 45 | 75 | 0.51 (0.33-0.78) | 0.002^*^ | 10 | 45 | 0.17 (0.08-0.36) | <0.001^*^ |
|  | Recessive | AG-GG | 325 | 314 | 1.00 |  | 170 | 169 | 1.00 |  |
|  |  | AA | 3 | 5 | 0.38 (0.09-1.69) | 0.204 | 0 | 7 | / | / |
|  | Log-additive | / | / | / | 0.54 (0.37-0.80) | 0.002^*^ | / | / | 0.19 (0.09-0.38) | <0.001^*^ |
| rs6543124 | Allele | T | 615 | 577 | 1.00 |  | 330 | 310 | 1.00 |  |
|  |  | A | 41 | 67 | 0.57 (0.38-0.86) | 0.009^*^ | 10 | 42 | 0.22 (0.11-0.45) | <0.001^*^ |
|  | Genotype | TT | 288 | 258 | 1.00 |  | 160 | 136 | 1.00 |  |
|  |  | AT | 39 | 61 | 0.56 (0.35-0.89) | 0.014^*^ | 10 | 38 | 0.20 (0.10-0.44) | <0.001^*^ |
|  |  | AA | 1 | 3 | 0.18 (0.02-1.81) | 0.143 | 0 | 2 | / | / |
|  | Dominant | TT | 288 | 258 | 1.00 |  | 160 | 136 | 1.00 |  |
|  |  | AA-AT | 40 | 64 | 0.54 (0.34-0.84) | 0.007^*^ | 10 | 40 | 0.20 (0.09-0.42) | <0.001^*^ |
|  | Recessive | AT-TT | 327 | 319 | 1.00 |  | 170 | 174 | 1.00 |  |
|  |  | AA | 1 | 3 | 0.19 (0.02-1.97) | 0.165 | 0 | 2 | / | / |
|  | Log-additive | / | / | / | 0.54 (0.35-0.83) | 0.005^*^ | / | / | 0.20 (0.10-0.42) | <0.001^*^ |

SNP: single nucleotide polymorphism; OR: odds ratio; CI: confidence interval.

*P* values were calculated by logistic regression analysis with adjusted.

**P*<0.05 represent statistical significance.

**Suppl_Table 3 The SNPs** **of *IL18R1* associated with susceptibility of chronic obstructive pulmonary disease in the subgroup tests (Age)**

| SNP | Model | Genotype | Age > 60 years | | | | Age ≤ 60 years | | | |
| --- | --- | --- | --- | --- | --- | --- | --- | --- | --- | --- |
|  |  |  | Case | Control | OR (95% CI) | *P* | Case | Control | OR (95% CI) | *P* |
| rs9807989 | Allele | T | 798 | 762 | 1.00 |  | 142 | 99 | 1.00 |  |
|  |  | C | 50 | 114 | 0.42 (0.30-0.59) | <0.001^*^ | 6 | 9 | 0.47 (0.16-1.35) | 0.182 |
|  | Genotype | TT | 377 | 336 | 1.00 |  | 68 | 45 | 1.00 |  |
|  |  | CT | 44 | 90 | 0.43 (0.27-0.69) | <0.001^*^ | 6 | 9 | / | / |
|  |  | CC | 3 | 12 | 0.14 (0.03-0.59) | 0.007^*^ | 0 | 0 | / | / |
|  | Dominant | TT | 377 | 336 | 1.00 |  | 68 | 45 | 1.00 |  |
|  |  | CC-CT | 47 | 102 | 0.39 (0.25-0.61) | <0.001^*^ | 6 | 9 | 0.78 (0.22-2.80) | 0.698 |
|  | Recessive | CT-TT | 421 | 426 | 1.00 |  | 74 | 54 | 1.00 |  |
|  |  | CC | 3 | 12 | 0.16 (0.04-0.66) | 0.011^*^ | 0 | 0 | / | / |
|  | Log-additive | / | / | / | 0.42 (0.28-0.62) | <0.001^*^ | / | / | 0.78 (0.22-2.80) | 0.698 |
| rs2287037 | Allele | C | 381 | 588 | 1.00 |  | 53 | 78 | 1.00 |  |
|  |  | T | 467 | 284 | 2.54 (2.09-3.09) | <0.001^*^ | 95 | 34 | 4.11 (2.43-6.95) | <0.001^**^ |
|  | Genotype | CC | 89 | 202 | 1.00 |  | 12 | 26 | 1.00 |  |
|  |  | TC | 203 | 184 | 2.14 (1.48-3.10) | <0.001^*^ | 29 | 26 | 1.97 (0.71-5.46) | 0.190 |
|  |  | TT | 132 | 50 | 5.49 (3.45-8.74) | <0.001^*^ | 33 | 4 | 11.40 (2.94-44.26) | <0.001^*^ |
|  | Dominant | CC | 89 | 202 | 1.000 |  | 12 | 26 | 1.00 |  |
|  |  | TT-TC | 335 | 234 | 2.84 (2.01-4.01) | <0.001^*^ | 62 | 30 | 3.45 (1.34-8.93) | 0.011^*^ |
|  | Recessive | TC-CC | 292 | 386 | 1.00 |  | 41 | 52 | 1.00 |  |
|  |  | TT | 132 | 50 | 3.52 (2.34-5.30) | <0.001^*^ | 33 | 4 | 7.85 (2.29-26.86) | 0.001^*^ |
|  | Log-additive | / | / | / | 2.32 (1.84-2.92) | <0.001^*^ | / | / | 3.12 (1.65-5.91) | <0.001^*^ |
| rs2058622 | Allele | A | 329 | 480 | 1.00 |  | 47 | 67 | 1.00 |  |
|  |  | G | 519 | 392 | 1.93 (1.59-2.34) | <0.001^*^ | 101 | 45 | 3.20 (1.92-5.34) | <0.001^*^ |
|  | Genotype | AA | 64 | 139 | 1.00 |  | 11 | 19 | 1.00 |  |
|  |  | GA | 201 | 202 | 1.90 (1.26-2.85) | 0.002^*^ | 25 | 29 | 1.46 (0.48-4.43) | 0.509 |
|  |  | GG | 159 | 95 | 3.07 (1.96-4.81) | <0.001^*^ | 38 | 8 | 6.66 (1.98-22.38) | 0.002^*^ |
|  | Dominant | AA | 64 | 139 | 1.00 |  | 11 | 19 | 1.00 |  |
|  |  | GG-GA | 360 | 297 | 2.27 (1.55-3.33) | <0.001^*^ | 63 | 37 | 2.78 (1.00-7.73) | 0.050^*^ |
|  | Recessive | GA-AA | 265 | 341 | 1.00 |  | 36 | 48 | 1.00 |  |
|  |  | GG | 159 | 95 | 1.99 (1.41-2.82) | <0.001^*^ | 38 | 8 | 5.29 (1.96-14.24) | 0.001^*^ |
|  | Log-additive | / | / | / | 1.74 (1.39-2.18) | <0.001^*^ | / | / | 2.66 (1.44-4.88) | 0.002^*^ |
| rs3771166 |  | G | 796 | 758 | 1.00 |  | 142 | 100 | 1.00 |  |
|  | Allele | A | 52 | 120 | 0.41 (0.29-0.58) | <0.001^*^ | 6 | 12 | 0.35 (0.13-0.97) | 0.048^*^ |
|  | Genotype | GG | 375 | 331 | 1.00 |  | 68 | 44 | 1.00 |  |
|  |  | AG | 46 | 96 | 0.40 (0.25-0.63) | <0.001^*^ | 6 | 12 | / | / |
|  |  | AA | 3 | 12 | 0.14 (0.03-0.57) | 0.006^*^ | 0 | 0 | / | / |
|  | Dominant | GG | 375 | 331 | 1.00 |  | 68 | 44 | 1.00 |  |
|  |  | AA-AG | 49 | 108 | 0.36 (0.23-0.56) | <0.001^*^ | 6 | 12 | 0.59 (0.17-2.03) | 0.401 |
|  | Recessive | AG-GG | 421 | 427 | 1.00 |  | 74 | 56 | 1.00 |  |
|  |  | AA | 3 | 12 | 0.16 (0.04-0.66) | 0.011^*^ | 0 | 0 | / | / |
|  | Log-additive | / | / | / | 0.39 (0.27-0.58) | <0.001^*^ | / | / | 0.59 (0.17-2.03) | 0.401 |
| rs6543124 | Allele | T | 801 | 783 | 1.00 |  | 144 | 104 | 1.00 |  |
|  |  | A | 47 | 99 | 0.46 (0.32-0.67) | <0.001^*^ | 4 | 10 | 0.29 (0.09-0.95) | 0.049^*^ |
|  | Genotype | TT | 378 | 347 | 1.00 |  | 70 | 47 | 1.00 |  |
|  |  | AT | 45 | 89 | 0.44 (0.28-0.71) | 0.001^*^ | 4 | 10 | / | / |
|  |  | AA | 1 | 5 | 0.09 (0.01-0.97) | 0.047^*^ | 0 | 0 | / | / |
|  | Dominant | TT | 378 | 347 | 1.00 |  | 70 | 47 | 1.00 |  |
|  |  | AA-AT | 46 | 94 | 0.42 (0.26-0.66) | <0.001^*^ | 4 | 10 | 0.55 (0.14-2.16) | 0.389 |
|  | Recessive | AT-TT | 423 | 436 | 1.00 |  | 74 | 57 | 1.00 |  |
|  |  | AA | 1 | 5 | 0.10 (0.01-1.09) | 0.058 | 0 | 0 | / | / |
|  | Log-additive | / | / | / | 0.42 (0.27-0.65) | <0.001^*^ | / | / | 0.55 (0.14-2.16) | 0.389 |

SNP: single nucleotide polymorphism; OR: odds ratio; CI: confidence interval.

*P* values were calculated by logistic regression analysis with adjusted.

**P*<0.05 represent statistical significance.

**Suppl_Table 4 The SNPs of *IL18R1* associated with susceptibility of chronic obstructive pulmonary disease in the subgroup tests (Smoking)**

| SNP | Model | Genotype | Smoking | | | | Non-smoking | | | |
| --- | --- | --- | --- | --- | --- | --- | --- | --- | --- | --- |
|  |  |  | Case | Control | OR (95% CI) | *P* | Case | Control | OR (95% CI) | *P* |
| rs9807989 | Allele | T | 409 | 386 | 1.00 |  | 531 | 475 | 1.00 |  |
|  |  | C | 27 | 46 | 0.55 (0.34-0.91) | 0.020^*^ | 29 | 77 | 0.34 (0.22-0.53) | <0.001^*^ |
|  | Genotype | TT | 193 | 173 | 1.00 |  | 252 | 208 | 1.00 |  |
|  |  | CT | 23 | 40 | 0.47 (0.26-0.85) | 0.013^*^ | 27 | 59 | 0.38 (0.23-0.63) | <0.001^*^ |
|  |  | CC | 2 | 3 | 0.85 (0.10-7.17) | 0.884 | 1 | 9 | 0.07 (0.01-0.53) | 0.011 |
|  | Dominant | TT | 193 | 173 | 1.000 |  | 252 | 208 | 1.00 |  |
|  |  | CC-CT | 25 | 43 | 0.48 (0.27-0.87) | 0.015^*^ | 28 | 68 | 0.33 (0.20-0.54) | <0.001^*^ |
|  | Recessive | CT-TT | 216 | 213 | 1.00 |  | 279 | 267 | 1.00 |  |
|  |  | CC | 2 | 3 | 0.95 (0.11-7.91) | 0.963 | 1 | 9 | 0.08 (0.01-0.62) | 0.016^*^ |
|  | Log-additive | / | / | / | 0.54 (0.32-0.93) | 0.025^*^ | / | / | 0.35 (0.22-0.55) | <0.001^*^ |
| rs2287037 | Allele | C | 199 | 286 | 1.00 |  | 235 | 380 | 1.00 |  |
|  |  | T | 237 | 148 | 2.30 (1.75-3.03) | <0.001^*^ | 325 | 170 | 3.09 (2.415-3.956) | <0.001^*^ |
|  | Genotype | CC | 47 | 90 | 1.00 |  | 54 | 138 | 1.00 |  |
|  |  | TC | 105 | 106 | 1.83 (1.13-2.97) | 0.014^*^ | 127 | 104 | 2.79 (1.83-4.28) | <0.001^*^ |
|  |  | TT | 66 | 21 | 6.95 (3.52-13.70) | <0.001^*^ | 99 | 33 | 7.21 (4.24-12.23) | <0.001^*^ |
|  | Dominant | CC | 47 | 90 | 1.00 |  | 54 | 138 | 1.00 |  |
|  |  | TT-TC | 171 | 127 | 2.58 (1.63-4.07) | <0.001^*^ | 226 | 137 | 3.84 (2.59-5.69) | <0.001^*^ |
|  | Recessive | TC-CC | 152 | 196 | 1.00 |  | 181 | 242 | 1.00 |  |
|  |  | TT | 66 | 21 | 4.82 (2.62-8.87) | <0.001^*^ | 99 | 33 | 4.10 (2.57-6.56) | <0.001^*^ |
|  | Log-additive | / | / | / | 2.46 (1.79-3.39) | <0.001^*^ | / | / | 2.70 (2.08-3.50) | <0.001^*^ |
| rs2058622 | Allele | A | 171 | 242 | 1.00 |  | 205 | 305 | 1.00 |  |
|  |  | G | 265 | 194 | 1.93 (1.48-2.53) | <0.001^*^ | 355 | 243 | 2.17 (1.71-2.77) | <0.001^*^ |
|  | Genotype | AA | 34 | 65 | 1.00 |  | 41 | 93 | 1.00 |  |
|  |  | GA | 103 | 112 | 1.64 (0.96-2.80) | 0.069 | 123 | 119 | 2.09 (1.31-3.32) | 0.002^*^ |
|  |  | GG | 81 | 41 | 4.03 (2.17-7.49) | <0.001^*^ | 116 | 62 | 3.63 (2.20-6.00) | <0.001^*^ |
|  | Dominant | AA | 34 | 65 | 1.00 |  | 41 | 93 | 1.00 |  |
|  |  | GG-GA | 184 | 153 | 2.22 (1.34-3.68) | 0.002^*^ | 239 | 181 | 2.62 (1.70-4.04) | <0.001^*^ |
|  | Recessive | GA-AA | 137 | 177 | 1.00 |  | 164 | 212 | 1.00 |  |
|  |  | GG | 81 | 41 | 2.87 (1.75-4.72) | <0.001^*^ | 116 | 62 | 2.25 (1.52-3.32) | <0.001^*^ |
|  | Log-additive | / | / | / | 2.02 (1.48-2.75) | <0.001^*^ | / | / | 1.89 (1.48-2.43) | <0.001^*^ |
| rs3771166 | Allele | G | 408 | 384 | 1.00 |  | 530 | 474 | 1.00 |  |
|  |  | A | 28 | 52 | 0.51 (0.31-0.82) | 0.007^*^ | 30 | 80 | 0.34 (0.22-0.52) | <0.001^*^ |
|  | Genotype | GG | 192 | 169 | 1.00 |  | 251 | 206 | 1.00 |  |
|  |  | AG | 24 | 46 | 0.41 (0.23-0.74) | 0.003^*^ | 28 | 62 | 0.37 (0.22-0.61) | <0.001^*^ |
|  |  | AA | 2 | 3 | 0.83 (0.10-7.03) | 0.868 | 1 | 9 | 0.08 (0.01-0.53) | 0.010^*^ |
|  | Dominant | GG | 192 | 169 | 1.00 |  | 251 | 206 | 1.00 |  |
|  |  | AA-AG | 26 | 49 | 0.43 (0.24-0.76) | 0.003^*^ | 29 | 71 | 0.32 (0.19-0.52) | <0.001^*^ |
|  | Recessive | AG-GG | 216 | 215 | 1.00 |  | 279 | 268 | 1.00 |  |
|  |  | AA | 2 | 3 | 0.95 (0.12-7.98) | 0.969 | 1 | 9 | 0.08 (0.010-0.62) | 0.016^*^ |
|  | Log-additive | / | / | / | 0.48 (0.29-0.82) | 0.007^*^ | / | / | 0.34 (0.22-0.53) | <0.001^*^ |
| rs6543124 | Allele | T | 413 | 402 | 1.00 |  | 532 | 485 | 1.00 |  |
|  |  | A | 23 | 40 | 0.56 (0.33-0.95) | 0.036^*^ | 28 | 69 | 0.37 (0.24-0.58) | <0.001^*^ |
|  | Genotype | TT | 196 | 182 | 1.00 |  | 252 | 212 | 1.00 |  |
|  |  | AT | 21 | 38 | 0.44 (0.23-0.81) | 0.009^*^ | 28 | 61 | 0.38 (0.23-0.64) | <0.001^*^ |
|  |  | AA | 1 | 1 | 1.90 (0.05-68.32) | 0.725 | 0 | 4 | / | / |
|  | Dominant | TT | 196 | 182 | 1.00 |  | 252 | 212 | 1.00 |  |
|  |  | AA-AT | 22 | 39 | 0.45 (0.25-0.84) | 0.011^*^ | 28 | 65 | 0.35 (0.21-0.59) | <0.001^*^ |
|  | Recessive | AT-TT | 217 | 220 | 1.00 |  | 280 | 273 | 1.00 |  |
|  |  | AA | 1 | 1 | 2.10 (0.06-73.23) | 0.683 | 0 | 4 | / | 0.999 |
|  | Log-additive | / | / | / | 0.49 (0.27-0.88) | 0.018^*^ | / | / | 0.35 (0.22-0.57) | <0.001^*^ |

SNP: single nucleotide polymorphism; OR: odds ratio; CI: confidence interval.

*P* values were calculated by logistic regression analysis with adjusted.

**P*<0.05 represent statistical significance.

**Suppl_Table 5 The SNPs of *IL18R1* associated with susceptibility of chronic obstructive pulmonary disease in the subgroup tests (Drinking)**

| SNP | Model | Genotype | Drinking | | | | Non-drinking | | | |
| --- | --- | --- | --- | --- | --- | --- | --- | --- | --- | --- |
|  |  |  | Case | Control | OR (95% CI) | *P* | Case | Control | OR (95% CI) | *P* |
| rs9807989 | Allele | T | 471 | 442 | 1.00 |  | 469 | 419 | 1.00 |  |
|  |  | C | 31 | 62 | 0.47 (0.30-0.74) | 0.001* | 25 | 61 | 0.37 (0.23-0.59) | <0.001* |
|  | Genotype | TT | 222 | 194 | 1.00 |  | 223 | 187 | 1.00 |  |
|  |  | CT | 27 | 54 | 0.43 (0.25-0.73) | 0.002* | 23 | 45 | 0.43 (0.25-0.76) | 0.003* |
|  |  | CC | 2 | 4 | 0.30 (0.05-1.80) | 0.188 | 1 | 8 | 0.09 (0.01-0.76) | 0.027* |
|  | Dominant | TT | 222 | 194 | 1.00 |  | 223 | 187 | 1.00 |  |
|  |  | CC-CT | 29 | 58 | 0.42 (0.25-0.70) | 0.001* | 24 | 53 | 0.38 (0.22-0.64) | <0.001* |
|  | Recessive | CT-TT | 249 | 248 | 1.00 |  | 246 | 232 | 1.00 |  |
|  |  | CC | 2 | 4 | 0.35 (0.06-2.05) | 0.243 | 1 | 8 | 0.10 (0.01-0.85) | 0.035* |
|  | Log-additive | / | / | / | 0.45 (0.28-0.73) | 0.001* | / | / | 0.40 (0.24-0.64) | <0.001* |
| rs2287037 | Allele | C | 234 | 339 | 1.00 |  | 200 | 327 | 1.00 |  |
|  |  | T | 268 | 165 | 2.35 (1.82-3.04) | <0.001* | 294 | 153 | 3.14 (2.42-4.09) | <0.001* |
|  | Genotype | CC | 54 | 113 | 1.00 |  | 47 | 115 | 1.00 |  |
|  |  | TC | 126 | 113 | 2.21 (1.44-3.41) | <0.001* | 106 | 97 | 2.44 (1.55-3.83) | <0.001* |
|  |  | TT | 71 | 26 | 5.58 (3.10-10.04) | <0.001* | 94 | 28 | 9.06 (5.11-16.04) | <0.001* |
|  | Dominant | CC | 54 | 113 | 1.00 |  | 47 | 115 | 1.00 |  |
|  |  | TT-TC | 197 | 139 | 2.82 (1.87-4.25) | <0.001* | 200 | 125 | 3.77 (2.48-5.72) | <0.001* |
|  | Recessive | TC-CC | 180 | 226 | 1.00 |  | 153 | 212 | 1.00 |  |
|  |  | TT | 71 | 26 | 3.48 (2.06-5.87) | <0.001* | 94 | 28 | 5.52 (3.32-9.17) | <0.001* |
|  | Log-additive | / | / | / | 2.34 (1.76-3.10) | <0.001* | / | / | 2.93 (2.22-3.88) | <0.001* |
| rs2058622 | Allele | A | 201 | 282 | 1.00 |  | 175 | 265 | 1.00 |  |
|  |  | G | 301 | 222 | 1.90 (1.48-2.44) | <0.001* | 319 | 215 | 2.25 (1.74-2.91) | <0.001* |
|  | Genotype | AA | 38 | 78 | 1.00 |  | 37 | 80 | 1.00 |  |
|  |  | GA | 125 | 126 | 1.90 (1.17-3.07) | 0.010* | 101 | 105 | 1.95 (1.19-3.18) | 0.008* |
|  |  | GG | 88 | 48 | 3.39 (1.95-5.89) | <0.001* | 109 | 55 | 4.40 (2.60-7.45) | <0.001* |
|  | Dominant | AA | 38 | 78 | 1.00 |  | 37 | 80 | 1.00 |  |
|  |  | GG-GA | 213 | 174 | 2.30 (1.45-3.64) | <0.001* | 210 | 160 | 2.75 (1.75-4.33) | <0.001* |
|  | Recessive | GA-AA | 163 | 204 | 1.00 |  | 138 | 185 | 1.00 |  |
|  |  | GG | 88 | 48 | 2.17 (1.41-3.35) | <0.001* | 109 | 55 | 2.87 (1.90-4.35) | <0.001* |
|  | Log-additive | / | / | / | 1.84 (1.39-2.42) | <0.001* | / | / | 2.11 (1.62-2.74) | <0.001* |
| rs3771166 | Allele | G | 469 | 441 | 1.00 |  | 469 | 417 | 1.00 |  |
|  |  | A | 33 | 67 | 0.46 (0.30-0.72) | <0.001* | 25 | 65 | 0.34 (0.21-0.55) | <0.001* |
|  | Genotype | GG | 220 | 191 | 1.000 |  | 223 | 184 | 1.00 |  |
|  |  | AG | 29 | 59 | 0.41 (0.24-0.68) | 0.001* | 23 | 49 | 0.39 (0.23-0.68) | 0.001* |
|  |  | AA | 2 | 4 | 0.30 (0.05-1.76) | 0.181 | 1 | 8 | 0.09 (0.01-0.75) | 0.026* |
|  | Dominant | GG | 220 | 191 | 1.000 |  | 223 | 184 | 1.00 |  |
|  |  | AA-AG | 31 | 63 | 0.40 (0.24-0.66) | <0.001* | 24 | 57 | 0.35 (0.20-0.59) | <0.001* |
|  | Recessive | AG-GG | 249 | 250 | 1.00 |  | 246 | 233 | 1.00 |  |
|  |  | AA | 2 | 4 | 0.35 (0.06-2.06) | 0.244 | 1 | 8 | 0.11 (0.01-0.86) | 0.036* |
|  | Log-additive | / | / | / | 0.43 (0.27-0.69) | <0.001* | / | / | 0.37 (0.23-0.60) | <0.001* |
| rs6543124 | Allele | T | 471 | 457 | 1.00 |  | 474 | 430 | 1.00 |  |
|  |  | A | 31 | 53 | 0.57 (0.36-0.90) | 0.017* | 20 | 56 | 0.32 (0.19-0.55) | <0.001* |
|  | Genotype | TT | 221 | 204 | 1.000 |  | 227 | 190 | 1.00 |  |
|  |  | AT | 29 | 49 | 0.50 (0.29-0.84) | 0.010* | 20 | 50 | 0.33 (0.19-0.59) | <0.001* |
|  |  | AA | 1 | 2 | 0.36 (0.03-4.37) | 0.421 | 0 | 3 | / | / |
|  | Dominant | TT | 221 | 204 | 1.00 |  | 227 | 190 | 1.00 |  |
|  |  | AA-AT | 30 | 51 | 0.49 (0.29-0.83) | 0.007* | 20 | 53 | 0.31 (0.17-0.54) | <0.001* |
|  | Recessive | AT-TT | 250 | 253 | 1.000 |  | 247 | 240 | 1.00 |  |
|  |  | AA | 1 | 2 | 0.40 (0.03-4.88) | 0.472 | 0 | 3 | / | / |
|  | Log-additive | / | / | / | 0.51 (0.31-0.84) | 0.008* | / | / | 0.31 (0.18-0.54) | <0.001* |

SNP: single nucleotide polymorphism; OR: odds ratio; CI: confidence interval.

*P* values were calculated by logistic regression analysis with adjusted.

**P*<0.05 represent statistical significance.

Suppl_Figure 1. The violin plot for the association of the genotypes with the mRNA expression of IL18R1 in the lung tissue. The width of the violin plot represents the density of the data points, i.e. the frequency with which a certain expression level occurs. The violin plot shows the median (a line running through the center of the violin plot) and the quartile (representing the upper, lower and middle ranges of the data distribution).
