# Supplementary figures and images for: Identification of genetic variants of the IL18R1 gene in association with COPD susceptibility
Source: Ann Med. 2025 Jan 23;57(1):2446690. doi: 10.1080/07853890.2024.2446690 (PMC11758794; doi:10.1080/07853890.2024.2446690)

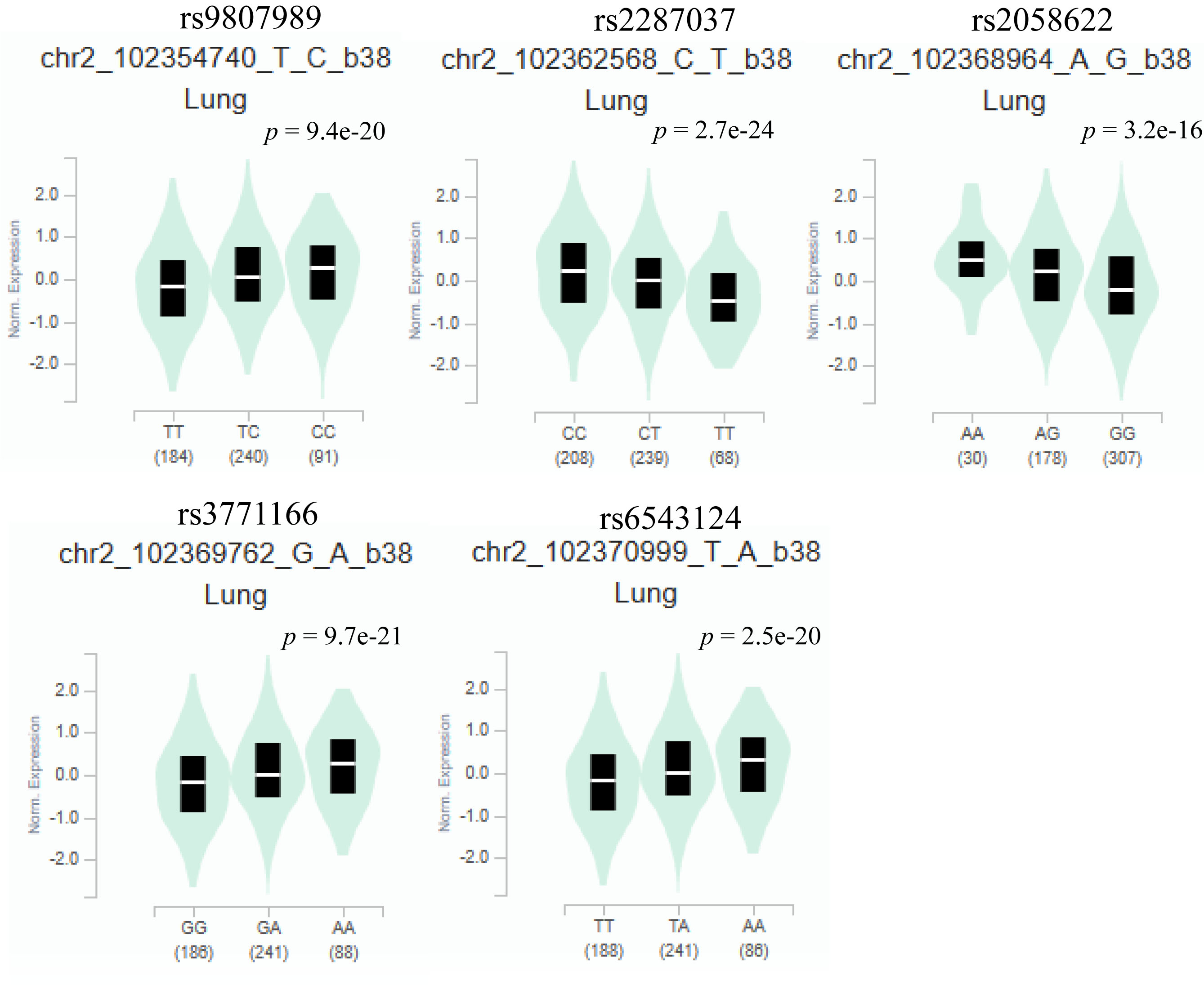

Supplement: Supplemental Material [file IANN_A_2446690_SM0540.zip › Suppl/Suppl_Figure 1.jpg]
